# Is the Link from Working Memory to Analogy Causal? No Analogy Improvements following Working Memory Training Gains

**DOI:** 10.1371/journal.pone.0106616

**Published:** 2014-09-04

**Authors:** J. Elizabeth Richey, Jeffrey S. Phillips, Christian D. Schunn, Walter Schneider

**Affiliations:** 1 Department of Psychology, Learning Research and Development Center, University of Pittsburgh, Pittsburgh, Pennsylvania, United States of America; 2 Department of Neurology, University of Pennsylvania, Philadelphia, Pennsylvania, United States of America; University of California, San Francisco, United States of America

## Abstract

Analogical reasoning has been hypothesized to critically depend upon working memory through correlational data [1], but less work has tested this relationship through experimental manipulation [2]. An opportunity for examining the connection between working memory and analogical reasoning has emerged from the growing, although somewhat controversial, body of literature suggests complex working memory training can sometimes lead to working memory improvements that transfer to novel working memory tasks. This study investigated whether working memory improvements, if replicated, would increase analogical reasoning ability. We assessed participants’ performance on verbal and visual analogy tasks after a complex working memory training program incorporating verbal and spatial tasks [3], [4]. Participants’ improvements on the working memory training tasks transferred to other short-term and working memory tasks, supporting the possibility of broad effects of working memory training. However, we found no effects on analogical reasoning. We propose several possible explanations for the lack of an impact of working memory improvements on analogical reasoning.

## Introduction

Analogical reasoning ability plays an important role in educational settings and can help determine students’ academic success [5]–[9]. As a result, students with poor analogical reasoning skills are likely to miss a number of the key concepts taught in class and in books using analogy. Why do some students successfully make and understand analogies while others do not? Memory, broadly defined, plays a key role in successful analogical transfer by influencing what prior experiences are retrieved [8], [10]. Clearly the organization of long-term memories matters for retrieval [11]–[13], but working memory has also been argued to play a role in the way mappings are made and inferences are drawn [2], [14]–[16]. Specifically, working memory capacity is thought to limit a person’s ability to keep track of all the information involved in the mapping between analogies, as well as the number of possible mappings between elements that a person can consider [17]. The large individual differences that exist in working memory capacity could then explain why individuals with similar content knowledge still differ in the success of their analogical reasoning.

If working memory differences are actually a key limitation that constrains analogical reasoning, a question naturally arises about whether these differences are addressable through educational interventions. Mnemonic devices, retrieval structures, and other memory tricks aside [18], working memory has long been regarded as a quality that varies in the population but imposes immutable limits on a learner. But a number of empirical studies have challenged this long-held view of working memory’s cognitive capacities, demonstrating large improvements on working memory tasks through training [19]–[22]. Most impressively, some studies have tested the transfer of such improvements to other, untrained working memory tasks and have found improvements there, too [19], [21], [23], [24]. Other recent work, however, has raised questions about whether working memory training consistently produces long-term changes in working memory capacity or transfer to other cognitive abilities [20], [25].

If there are forms of training that truly produce general changes to working memory capacity, then this line of research presents powerful theoretical and practical opportunities for new contributions to the robust literature exploring analogical reasoning. On a theoretical level, an experimental manipulation of working memory permits a closer exploration of its direct effects on analogical reasoning, providing an opportunity to test whether working memory capacity is indeed a key bottleneck to analogical reasoning. On a practical level, it suggests a training intervention that could improve students’ analogical reasoning and potentially bolster learning. In the following sections, we review existing evidence of the relationship between working memory and analogical reasoning and the behavioral effects of working memory training.

### Working memory and analogical reasoning

The process of analogical reasoning hinges on two components: retrieval of an appropriate analog (or encoding of the analog, if it is provided) and mapping between the features of the two analogs [13], [26]. Mechanistically, analogical reasoning theories have highlighted the importance of successfully mapping structural features between analogs. The mapping process requires a learner to keep track of both the features that are being mapped across analogs and the relationships among the features within a given analog. Working memory is responsible for holding on to all pieces of information being manipulated at a given time, so the task of mapping analogs should place a large burden on working memory. For this reason, working memory capacity is believed to play an important role in successful analogical reasoning.

In many cases, the final step of analogical problem solving involves applying the inferred relation from the base analog to a set of possible solutions, either in the form of provided solutions or generated solutions within a search space. Sternberg [27] found that a simple model that excluded the application phase could account for observed process data; however, all of his process data was conducted on complete analogies, with participants instructed to judge whether they were true or false. Many analogical problems in the real world do not include solutions, and several lines of research suggest that working memory is particularly important when the solution component is not provided in the problem. Unsworth and Engle [28] emphasize working memory’s role in retrieving information outside of immediate awareness, and Wiley and Jarosz [29] stress the importance of working memory for controlling attention while ignoring distractions within a problem space. This perspective is consistent with Wiley, Jarosz, Cushen, and Colflesh’s [30] view that it is the access and generation of new rule combinations, as opposed to the number of rules being held in the mind at once, that explains the relationship between working memory capacity and performance on analogical reasoning tasks. If working memory plays a critical role in the recall of a piece of information not at the forefront of the mind, be it a rule combination or a possible analog, then analogy tasks requiring participants to generate their own analogs should show a greater working memory effect than tasks that provide analog choices. This research has important implications for the choice of tasks to assess the relationship between analogical reasoning and working memory, which we discuss in greater detail below.

Several major studies have found a strong correlational relationship between working memory and tasks that measure analogical reasoning. For example, Kyllonen and Christal [1] found that participants’ (*N* = 399) verbal analogy task performance was strongly, positively correlated with performance on three of four working memory tasks, while the correlation with the fourth, digit span, was moderately strong (*r* in the range of .30 to .40). There have also been several experimental investigations of the role of working memory capacity in analogical reasoning, with results showing that verbal and spatial distractor tasks introduced to consume working memory reduce success on verbal and spatial analogy tasks, respectively [2], [16]. Looking at working memory’s role in the process of identifying deep, relational structures, Waltz et al. [2] found that working memory distracter tasks reduced the likelihood that participants would identify relational matches across analogical images (i.e., the same relationship between two objects appears in both pictures) and increased the likelihood that they would identify object matches (i.e., the same object appears in both pictures). However, such dual task manipulations may have changed attention rather than working memory capacity.

Building on the large body of literature investigating the mechanisms of analogical reasoning and the evidence of a possible connection between analogical reasoning and working memory capacity, we now turn to research aimed at changing working memory capacity. This line of work suggests an experimental paradigm for rigorous testing of the relationship between working memory and analogical reasoning, with the potential for practical applications aimed at improving learners’ analogical reasoning.

### Changing working memory

A. B. Morrison and Chein [20] characterize working memory training as falling into two groups: “strategy training,” which focuses on increasing the amount of information held in working memory through the use of strategies like rehearsal and elaborative encoding, and “core training,” which focuses on improving capacity or speed of domain-general working memory through adaptive, multi-modal tasks requiring rapid encoding and retrieval. Strategy training is typically domain-specific and hard to simultaneously apply during problem solving and analogical reasoning. Core training often takes the form of a variant of the *n-*back task, in which participants indicate whether a stimulus is identical to one presented *n* items earlier; the updating task, which exposes participants to a stream of items and prompts them at unpredictable points to recall the most recent *n* items in order; or the complex working memory task, which requires participants to store and eventually retrieve one stream of stimuli while making intermittent judgments about another set of stimuli.

Klingberg [19] and A. B. Morrison and Chein [20] reviewed a number of studies that showed evidence of performance improvements on working memory tasks following training in a variety of populations (e.g., college students, children, children with ADHD, older adults). These reviews noted that studies have shown relatively consistent success extending performance changes to untrained working memory tasks such as span tasks and updating tasks. Based on a rigorous meta-analysis examining only experiments and quasi-experiments with a control group, Melby-Lervåg and Hulme [22] also concluded that there was robust evidence of immediate transfer to novel visuospatial and verbal working memory tasks, with moderate-to-large gains on both types of tasks. Looking at whether effects were retained after a delay, the authors found a small effect on visuospatial working memory tasks (average delay of five months) and no effect on verbal working memory tasks (average delay of nine months). Given the standards for inclusion in the meta-analysis, some of the categories examined had few studies (e.g., only four were included in the test of delayed verbal transfer), highlighting the need for further investigation of these questions using rigorous experimental design.

Although some noteworthy experiments have failed to replicate working memory training effects [25], these reviews of the literature suggest a generally consistent pattern of findings regarding the short-term transfer of working memory training to other measures of working memory. Although there is great variability across experiments in terms of population, training task, dose, and experimental design, there is only modest evidence that any of these factors explains the differences found across studies. Testing the aforementioned variables, Melby-Lervåg and Hulme [22] found that only participants’ age moderated immediate transfer effects on verbal working memory tasks, while training task type moderated immediate transfer effects on visuospatial working memory tasks.

### Transfer of working memory training to other tasks

On the whole, there have been inconsistent results regarding whether working memory training improves performance on tasks measuring various components of general intelligence [19]–[21]. Several working memory training studies have found performance improvements on variations of the Raven’s Progressive Matrices task [23], [31]–[35], while others have failed to find transfer effects [3], [25], [36], [37]. Melby-Lervåg and Hulme [22] examined far-transfer measures of verbal and nonverbal ability, attentional inhibition, word decoding, and arithmetic and found a significant mean effect size only on attentional inhibition measures (i.e., the Stroop task).

The absence of significant working memory training effects on far transfer measures might suggest that it is a poor approach for testing whether changes in working memory produce changes in analogical reasoning. However, as Redick et al. [25] argue, many of the far transfer measures assessed in working memory paradigms are presented without a strong theoretical explanation of the possible mechanisms that might create transfer effects. Consequently, it is difficult to differentiate in the literature between far transfer failures occuring because of a lack of working memory training effects, a weak or absent relationship between working memory and the far transfer construct of interest, or some other misalignment between theeoretical constructs and measures. We address this issue by clearly explicating the theoretical relationship between working memory and the processes of analogical reasoning, and by selecting analogical reasoning tasks that have been associated (correlationally) with working memory in prior work.

### Present study

In the present work, we sought to test the relationship between working memory and analogical reasoning by experimentally manipulating working memory through training. We first attempted to replicate other results showing that participants who completed working memory training would demonstrate significant improvement on a battery of working memory measures. This replication is important given the controversy over training effects even on immediate, near-transfer measures of working memory. Further, there can be no meaningful test of transfer to analogical reasoning without first showing robust working memory effects. We then tested whether participants in the working memory-training group would perform better on two analogical reasoning tasks that have previously been associated with working memory constraints.

## Methods

### Participants

Twenty-five participants (age 18–30) from the University of Pittsburgh community were paid by the hour and received performance and completion bonuses. A separate group of 29 participants were recruited into a control condition that completed the same pre and posttest measures of working memory and analogical reasoning. As a result of time limitations, some control participants failed to complete all of the tests. Three did not complete the spatial working memory test, six did not complete the verbal analogy test, and two did not complete the visual analogy test. These participants are excluded from analyses concerning the tests they did not complete but included in other analyses. These *N*s provide a power of .67 and .74 to find moderate gain effects (*d* = 0.5) within the training and control groups, respectively. The University of Pittsburgh’s Institutional Review Board approved the research, and all participants provided informed, written consent before participating.

### Procedure

Participants in the working memory training group first completed pretest measures targeting working memory and a verbal analogy task. Two working memory tasks – one verbal and the other spatial – were included to test the generality of the working memory training effects. During the same session, participants completed their first training session. Participants returned for nine additional training sessions. Following their final training session, participants completed the same two working memory tasks and the verbal analogy task administered at pretest, as well as a visual analogy task. Participants in the control condition completed the pretest and posttest measures in two sessions. Given the limited number of visual analogy items available and the saliency of each one, the visual analogy test was given only as a post-training measure to both conditions.

### Materials

#### Training materials

Training tasks were administered on a computer using E-Prime 2.0. The complex working memory (CWM) training consisted of verbal and spatial tasks [3], [4], which were completed in two separate blocks. Repeated practice with the CWM tasks has been shown to produce improvements in working memory [21], although it is important to note that the statistical strength of that demonstration was marginal, and thus replicating the results is especially important. Both the verbal and spatial working memory tasks included a memory component and decision component.

**Figure 1:**
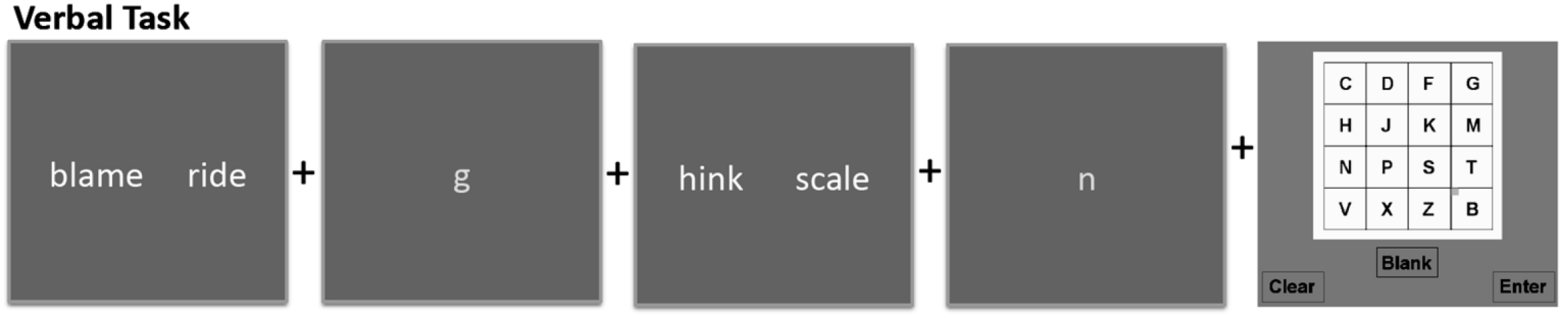
Sample series of stimuli for the verbal CWM task.

In the verbal trials, participants were shown a series of individual letters to remember. Between each letter, participants were shown two side-by-side strings of letters and instructed to judge whether both strings matched in terms of being words or non-words. At the end of the series, participants were prompted to input the letters they saw in the order they were shown on a 4×4 grid containing both the target letters and distracter letters (Figure 1).

**Figure 2:**
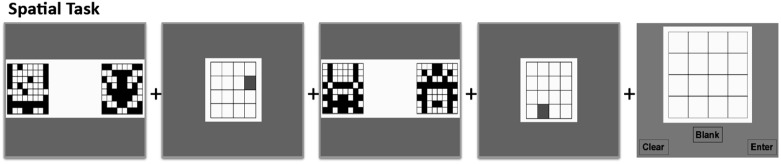
Sample series of stimuli for the spatial CWM task.

The spatial portion of the CWM training followed the same design as the verbal task. Participants saw a series of spatial locations highlighted in red on a 4×4 grid. Participants were instructed to remember the locations in the order presented. In between the presentation of each grid stimulus, participants performed a decision task in which they judged whether a pair of patterns in black boxes matched in symmetry. At the end of the series, participants were prompted to input the red square locations they had seen in the order in which they were shown on a 4×4 grid of blank squares (Figure 2).

For both working memory tasks, participants received feedback about their accuracy after every trial. Task demands were adaptive and became easier or more difficult based on performance. The first trial included four memory stimuli and four decision tasks; two consecutive successful trials resulted in a one-item set increase, while two consecutive unsuccessful trials resulted in a one-item set decrease. Participants’ accuracy was reported as the maximum sequence length successfully recalled. A day of practice lasted 40 minutes and was split evenly between spatial and verbal tasks; on average, participants completed 26 verbal trials and 40 spatial trials per session.

#### Pretest and posttest materials

Pretest and posttest measures included a digit span task, spatial working memory task, and verbal analogy task. The posttest also included a visual analogy task.

#### Digit span

The digit span task was selected as a test of working memory because it is commonly used as a measure of working memory capacity that has been associated with analogical reasoning [1] and has shown training effects on working memory in past work [23], [32], [37], [38]. It is also quite different from both training tasks in terms of structure and stimuli content. Critically, it specifically targets storage capacity, so any effects on digit span would indicate a change to storage capacity as opposed to familiarity with the training task or change to some other construct associated with working memory (e.g., task management). In this task, participants were presented with audio stimuli in the form of single-digit numbers presented at a rate of one number every second. The number of digits presented in a sequence started at three and increased by a single digit when participants accurately recalled the previous sequence of numbers across two consecutive trials. If participants made errors in two consecutive trials, the task ended. Participants were assigned a digit span representing the maximum number of digits accurately recalled in a single sequence.

#### Spatial working memory

**Figure 3:**
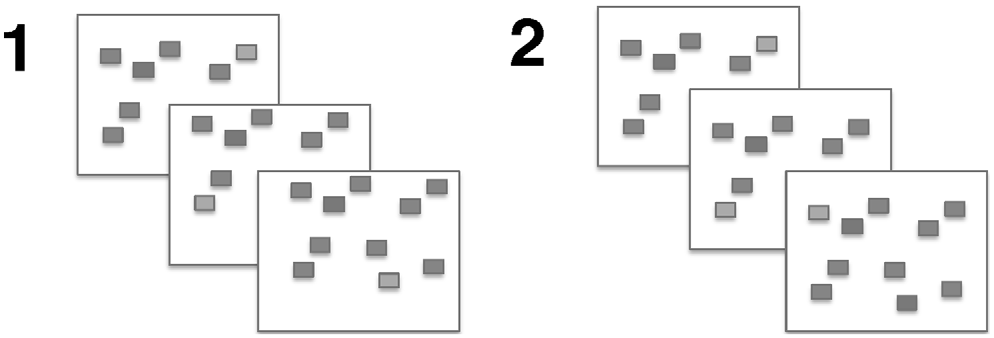
Sample of three trials from the spatial working memory pre/post task.

Gmeindl, Walsh, and Courtney’s [39] spatial working memory task was included to test the generality of spatial working memory training. On a screen with ten blue blocks, participants viewed a sequence of different blocks lighting up. They then viewed a second sequence and were asked to judge whether the second sequence was identical to the first (Figure 3). Sequence length was not adaptive. All participants completed a total of 24 trials, with eight trials each at set sizes four, six, and eight items.

#### Verbal analogy task

The verbal analogy task employed 80 verbal analogies taken from Green et al. [40], because of the large set of items that are normed for item difficulty and semantic distance. Participants in Green et al. [40] assessed whether complete analogies were true or false, and they performed at high accuracy. Prior work suggests that an analogical reasoning task focused on capturing the effects of working memory capacity should be open-ended rather than multiple-choice, as this may increase the role of working memory in the solution process [e.g., 28]. An analogy task that includes response options captures the working memory demands of the mapping and inference steps of analogy, but it does not capture the additional working memory demands of retrieval.

To increase analogy task difficulty, we removed the fourth element of the analogies and presented them in the form of “A:B::C:?”, with participants instructed to generate the correct response. To avoid repetition of stimuli between pretest and posttest, the list was divided into two sets of 40 items balanced for semantic distance and difficulty. Participants’ accuracy and reaction times for each verbal analogy were calculated.

#### Visual analogy task

**Figure 4:**
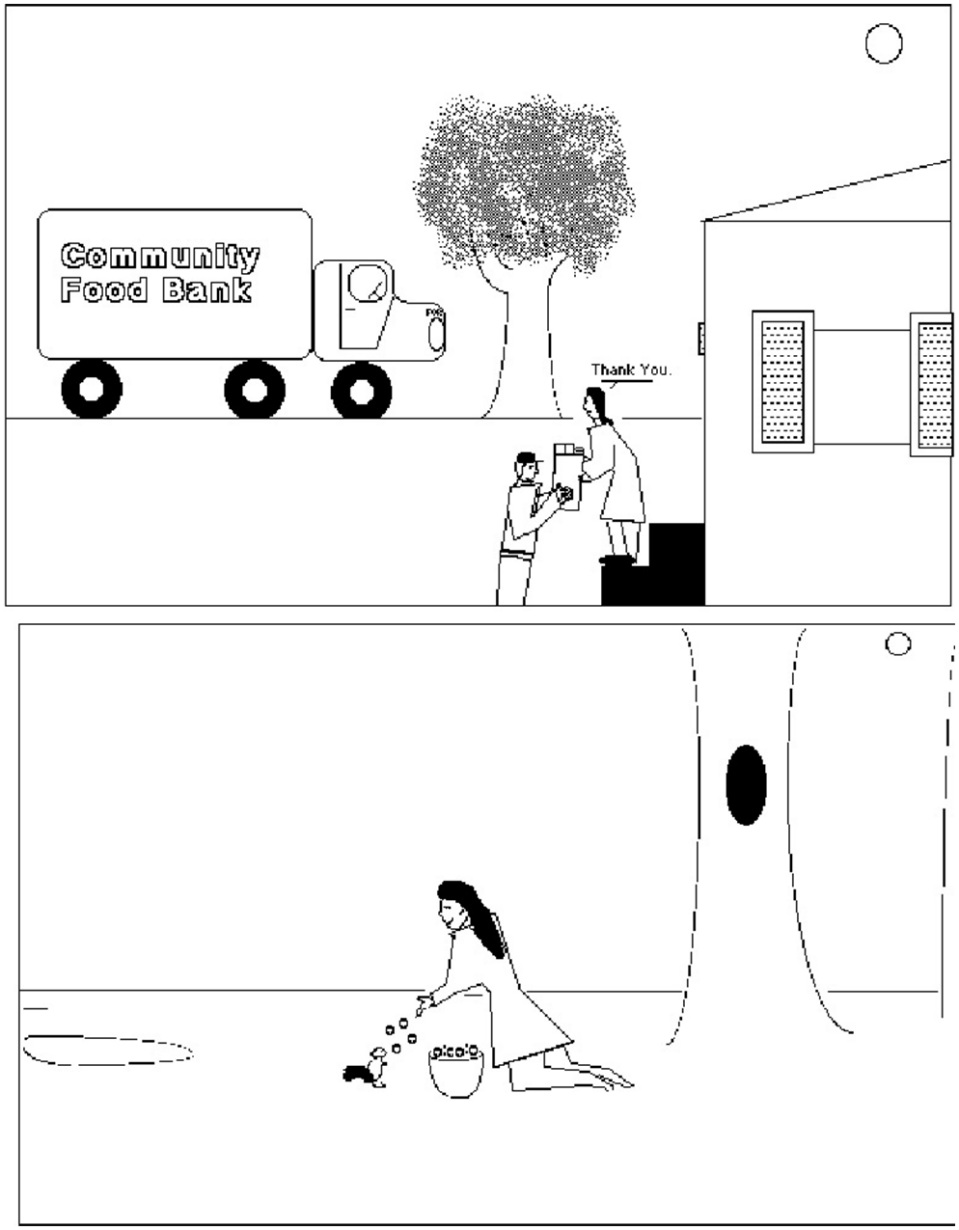
Sample image pair containing an object match and a relational match. Participants were instructed to click on the analogous item in the lower image.

The posttest also included a visual analogy task. For this task, eight pairs of pictures were displayed one at a time on a computer screen, with pictures stacked vertically. Images were taken from Markman and Gentner [41] and were designed to contain both an object match (i.e., the selected items appear in both images) and a relational match (i.e., the selected items have the same relationship with other items in the images). Figure 4 shows one of the picture sets used; in this example, the woman in the upper image is the target. The woman in the lower image is the object match and the squirrel in the lower image represents the relational match because it, like the woman, is receiving food.

## Results

### Training task improvements

Changes in training task performance were calculated by comparing Day 1 task accuracy against Day 10 task accuracy. A paired-sample t-test comparing performance on the spatial CWM task at the beginning of the training (*M* = 3.08, *SD* = 0.70) and the end of the training (*M* = 6.44, *SD* = 1.33) revealed a large improvement across scores, *t*(24) = 13.05, *p*<.01, *d* = 3.16. Twenty-one of the 25 participants’ scores increased by at least 3 points.

**Figure 5:**
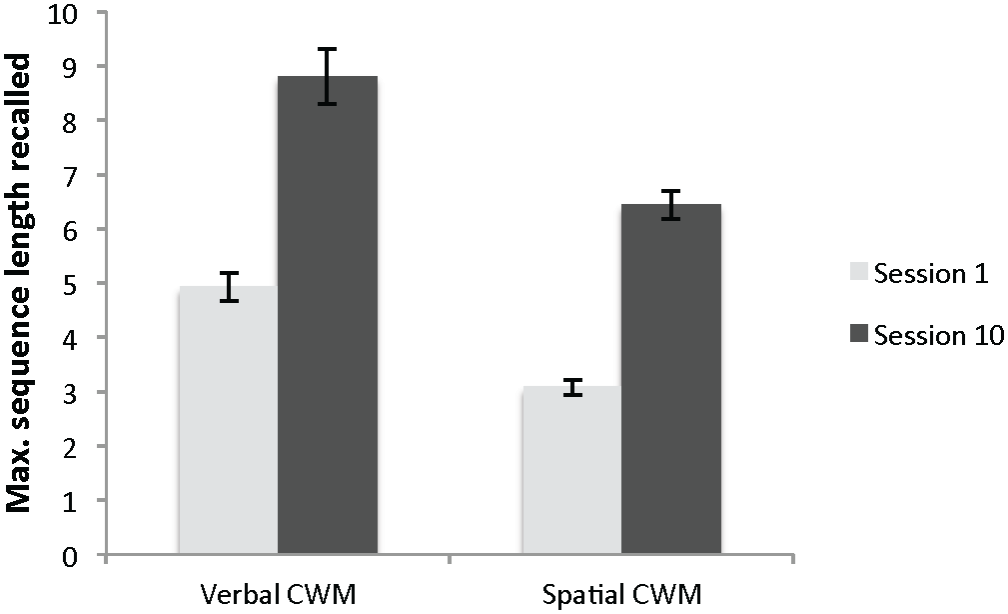
Mean verbal and spatial CWM performance on the first and last sessions of the intervention with SE bars.

A paired-sample t-test comparing participants’ performance on the verbal CWM task at the beginning of the training (*M* = 4.92, *SD* = 1.29) and the end of the training (*M* = 8.80, *SD* = 2.53) also revealed a large improvement across scores, *t*(24) = 8.07, *p*<.01, *d* = 1.93. Sixteen of the 25 participants’ scores increased by at least 3 points (Figure 5).

### Working memory transfer task performance

#### Digit span

**Figure 6:**
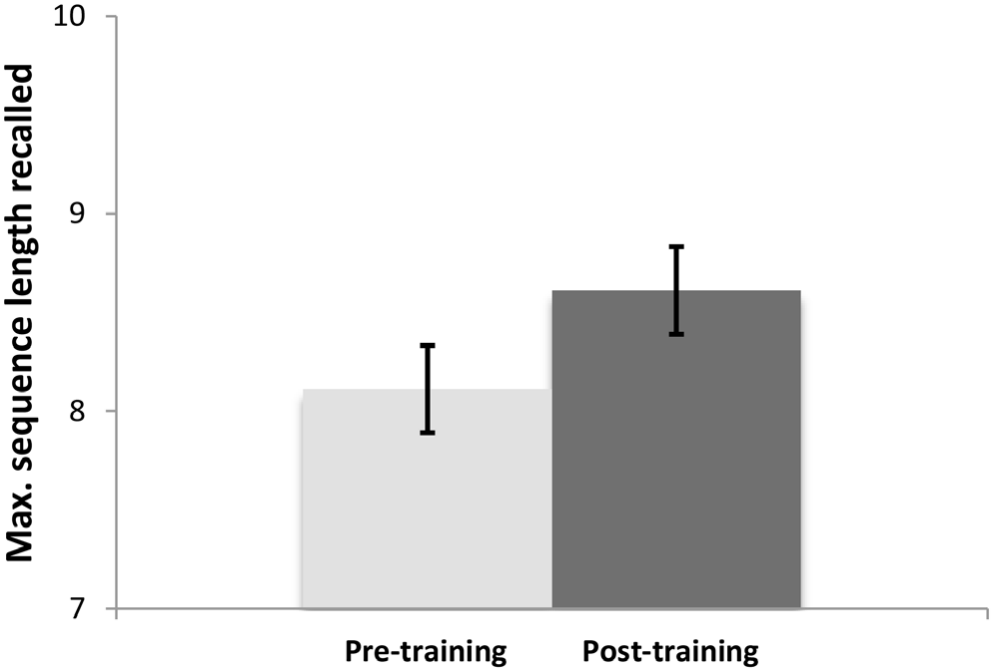
Intervention participants’ mean digit span performance pre- and post-training with SE bars.

Seven training group participants’ digit span performance data were excluded because of experimenter error (i.e., starting participants at eight instead of three on either the pretest or posttest). Training group participants’ performance on the digit span task significantly improved from pre (*M* = 8.11, *SD* = 0.58) to post (*M* = 8.61, *SD* = 0.85), *t*(17) = 2.47, *p* = .02, *d* = 0.69 (Figure 6). By comparison, the control group’s performance did not change significantly from pre (*M* = 7.72, *SD* = 1.19) to post (*M* = 8.07, *SD* = 1.19), *t*(28) = 1.8, *p* = .12, *d* = 0.29. Thus complex working memory training generalized to a simpler span measure using other stimuli, suggesting that the training effect involved storage capacity rather than stimulus chunking effects or complex task management.

#### Spatial working memory

**Figure 7:**
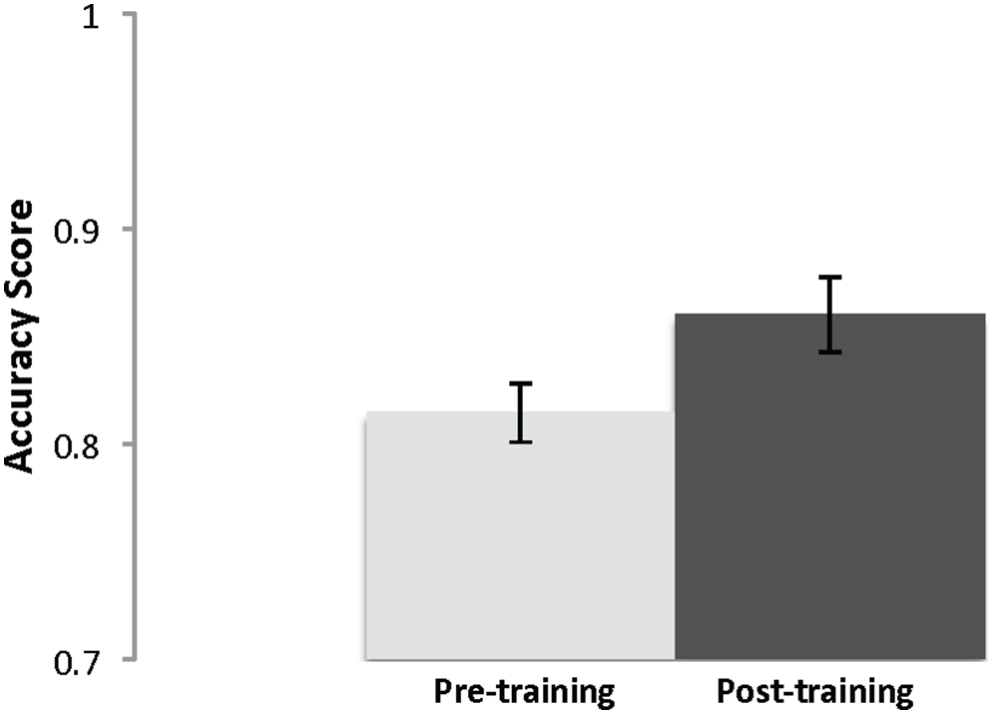
Intervention participants’ mean spatial working memory performance pre- and post-training with SE bars.

Training group participants’ performance on the spatial working memory task also improved significantly from pre (*M* = .81, *SD* = .07) to post (*M* = .86, *SD* = .09), *t*(24) = 2.37, *p* = .03, *d* = 0.62 (Figure 7). Reaction time in milliseconds from pre-training (*M* = 518, *SD* = 239) to post-training (*M* = 519, *SD* = 197) did not change, *t*(24) = .03, *p*<.01, suggesting that the accuracy improvements were not the result of a speed-accuracy shift. By comparison, the control group’s performance did not change significantly from pre (*M* = .81, *SD* = .08) to post (*M* = .83, *SD* = .07), *t*(25) = 1.22, *p* = .23, *d* = 0.27. Again, this demonstrates the generalizability of the training effects to an untrained measure of working memory, this time targeting spatial components.

### Analogy transfer task performance

#### Verbal analogy

**Figure 8:**
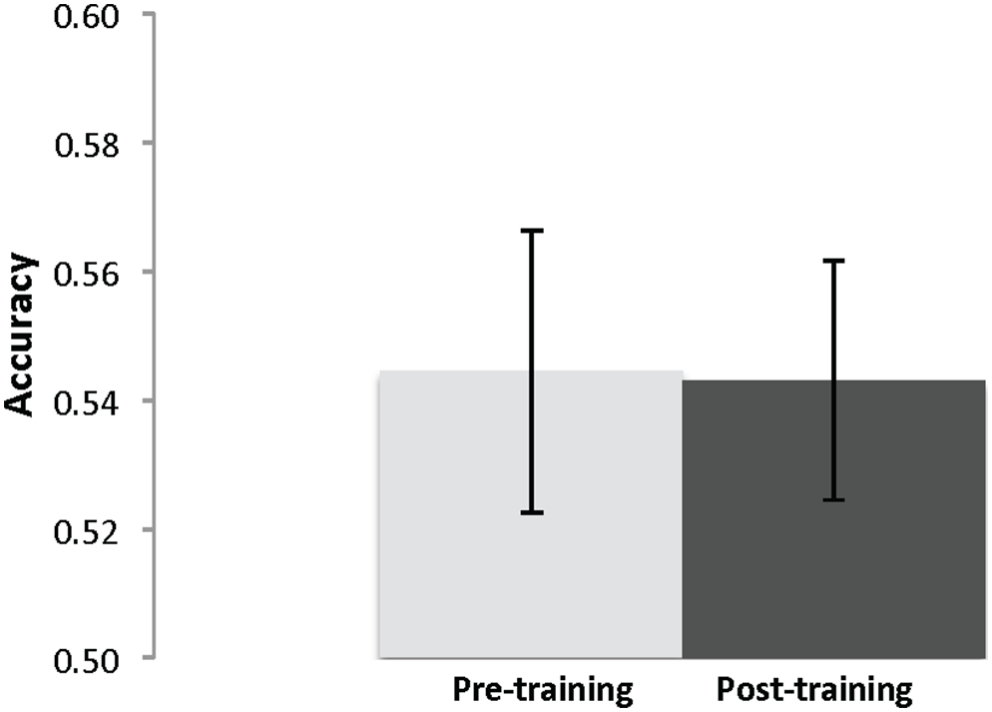
Intervention participants’ mean verbal analogy task performance pre- and post-training with SE bars.

Accuracy on the two versions was equivalent and so data was collapsed across versions. Accuracy on the verbal analogy task was essentially the same before (*M* = .55, *SD* = .11) and after training (*M* = .54, *SD* = .08) on a paired t-test, *t*(24) = .15, *p* = .88, *d* = 0.10 (Figure 8), as was average solution time per item in seconds from pre-training (*M* = 9.69, *SD* = 3.05) to post-training (*M* = 9.60, *SD* = 2.75), *t*(24) = .17, *p* = .87, *d* = 0.03. Similarly, the control group’s performance did not change significantly from pre (*M* = .52, *SD* = .10) to post (*M* = .53, *SD* = .09), *t*(23) = .49, *p* = .63, *d* = 0.11, nor did their solution time from pre (*M* = 9.77, *SD* = 3.30) to post (*M* = 8.76, *SD* = 3.4), *t*(23) = 1.59, *p* = .13, *d* = 0.30.

When focusing only on training participants with verbal CWM score improvements of three or more, performance on the verbal analogy task was again the same before (*M* = .57, *SD* = .09) and after training (*M* = .57, *SD* = .07), *t*(15) = .07, *p* = .95, *d*<0.01. Using only participants who experienced a gain on digit span of at least one standard above the mean, there were also no changes in verbal analogy performance before (*M* = .50, *SD* = .00) and after training (*M* = .50, *SD* = .14), *t*(1) <.01, *p*>.99, *d*<0.01.

#### Visual analogy

Because the visual analogies were provided only as a post-test measure, the statistical comparison on this task is to the control condition participants who completed the visual analogy task without doing any training. An ANOVA revealed no significant difference between CWM training participants and control participants in accuracy, (CWM *M* = .77, *SD* = .22; control *M* = .76, *SD* = .17), *F*(1, 50) = 0.04, *p* = .84, *d* = 0.05, and no difference in solution times in seconds (CWM *M* = 98.2, *SD* = 19.3; control *M* = 102, *SD* = 25.3), *F*(1, 50) = .54, *p* = .47, *d* = 0.17. Similar lack of gains were found when focusing on training participants who experienced a pre- to post-intervention spatial CWM score improvement of three or more (CWM *M* = 0.79, *SD* = 0.18; control *M* = 0.76, *SD* = 0.17), *F*(1, 46) = .26, *p* = .61, *d* = 0.17, or when focusing on participants who experienced a gain on the spatial working memory transfer task of at least one standard deviation above the mean (CWM *M* = 0.58, *SD* = 0.44; control *M* = 0.76, *SD* = 0.18), *F*(1, 28) = 2.1, *p* = .17, *d* =  −0.54; note that this effect trending toward statistical significance is in the opposite direction.

#### Correlations of WM and analogy task performance

Past studies using these analogy tasks provided some data to suggest that working memory was implicated in the tasks. Table 1 presents correlations between working memory measures and analogy measures.

**Table 1:** Pearson correlations between analogy performance and performance on pre-training WM transfer measures.

	Pre-training digit span	Pre-training spatial WM
Pre-training verbal analogy accuracy	.46†	.27
Visual analogy accuracy	.19	.51**

† *p*<.10,

*p<.05,

**p<.01.

We see that there are some large correlations: digit span predicts verbal analogy performance and spatial working memory predicts visual analogy performance. This pattern is consistent with expectations based on the model of separate visuospatial and phonological components of working memory [42]. Overall, these results show that we have sufficient precision in our analogy performance measures to detect relationships with working memory capacity, and, more generally, that we have selected the kind of analogy and working memory tasks that appear related to one another.

Finally, we tested whether improvements in working memory performance on any measures predicted post-training performance on either visual or verbal analogy tasks (Table 2). Despite the strong correlations between pre-training working memory measures and analogy task performance and the significant transfer effects of working memory training to other working memory measures, no changes in working memory performance were associated with post-training analogy task performance. This provides evidence that although the training effects transferred to other working memory measures, they did not transfer to analogical reasoning measures.

**Table 2:** Pearson correlations between post-training analogy task performance and changes in performance on working memory measures from before to after the training.

	Pre- to post change in digit span	Pre- to post change in spatial WM	Session 1 to 10 change in Verbal CWM	Session 1 to 10 Change in Spatial CWM
Post-training verbal analogy accuracy	.09	.02	.16	.01
Post-training visual analogy accuracy	.17	−.07	−.01	.07

† *p*<.10,

*p<.05,

**p<.01.

## Discussion

Although the results demonstrate improvement across multiple working memory assessments following a relatively limited training intervention, we did not find any change in verbal analogy performance as a result of the working memory training, nor did we find a training advantage for participants’ visual analogy performance compared to a control condition that did not receive the working memory training. Furthermore, the results replicate past findings associating individual differences in working memory measures with analogy task performance, providing evidence that there was sufficient power in the measures to detect a correlation between constructs.

Results from another training study using the same pool of participants and the same pre-post working memory tasks can be used to rule out simple test-retest effects on the working memory transfer measures. In this study, 23 participants who were recruited using the same criteria and had similar baseline performance on all measures received training focused on motor skills, specifically in the form of a serial reaction task that required a similar level of attention to the CWM training but did not involve working memory (see [43] for details of the study). The training spanned the same number of days and had the same duration as the CWM training study, and thus pre-post conditions are exactly matched. The training produced a large gain (*d* = 1.76) in performance on the serial reaction task but produced no changes in spatial working memory accuracy or reaction time (*p* = .83, *d* = 0 and *p* = .25, *d* = 0.27), and a non-significant gain in the digit span task (*p* = .11, *d* = 0.39). Thus, while there is a trend toward some test-retest effects on the digit span task, overall the working memory transfer results of the CWM training cannot be attributed to test-retest effects.

Taken together, these data suggest that the absence of a training effect on analogy task performance was not due to the absence of a general, transferable working memory training effect, nor was it due to insufficient power in the measures to detect a relationship between working memory capacity and analogical reasoning. From a practical perspective, the results provide further evidence that the transfer effects of working memory training may be too limited to affect performance on more distant transfer tasks that nevertheless are associated with working memory capacity. Such an interpretation is consistent with the conclusions drawn from the recent meta-analytic review investigating transfer effects of working memory training [20].

Several possible explanations exist for the lack of a significant transfer effect on richer analogy tasks. First, it is likely that the magnitude of transfer effects diminish as the distance of the transfer measures increase. The effect sizes of the complex working memory training on the trained tasks themselves were quite large (*d* = 2.84 for spatial CWM and *d* = 1.78 for verbal CWM), while the effect sizes on the transfer working memory measures were roughly a third as large (*d* = 0.58 for spatial working memory and *d* = 0.69 for digit span). Consequently, it may be that a significantly larger initial training effect – one that would likely arise only from more extended training – would be required to produce the more distant transfer effects measured with the analogy tasks. However, a 0.5 effect size improvement in working memory performance is no trivial change and should have produced benefits for other tasks that have a working memory capacity bottleneck, if such benefits could be realized through working memory training. Furthermore, Melby-Lervåg and Hulme [22] found no evidence that working memory training dosage moderates transfer outcomes. Instead, we propose that the best test of whether the number of working memory training sessions was sufficient is whether or not the training sessions led to improvements on untrained measures of working memory, which they did.

A second possible explanation is that working memory is only indirectly associated with analogy performance, rather than being a critical bottleneck. Although some working memory is clearly required for analogical mapping and inference, it may be that a small amount is sufficient and that further increases do not affect analogical performance. On the other hand, general knowledge has also been associated with analogical performance, such as Kyllonen and Christal’s [1] finding that verbal analogy performance and word knowledge (*r* = .34) were about as strongly correlated as verbal analogy performance and digit span (*r* = .36). Previously observed correlations might reflect working memory’s supporting role in general knowledge acquisition, which in turn may improve performance on analogical reasoning tasks. Comparing an open-ended analogical reasoning task with a multiple-choice analogical task in a different domain, Novick and Holyoak [44] found that success on the open-ended tasks was correlated not with success on the other analogy measures but rather with performance on other measures of the open-ended task domain.

Third, it may take time for people to learn how to use high working memory capacity in various problem-solving applications, including analogy. For example, it may be that analogies can be solved through various strategies, some creating higher working memory demands and some creating lower working memory demands. In providing working memory *capacity* training, we did not provide training in how to use problem-solving strategies that take advantage of higher working memory capacity. Individuals normally discover over extended task experiences which strategies best fit their skills, and those strategy choices change over time as skills and capacities change [45], [46]. Beilock and DeCaro [47] demonstrated the association of strategy sophistication with working memory constraints as well as participants’ lack of ability to quickly adapt strategy use.

We believe these latter two explanations are more consistent with the available data and should be explored in future research. Under the weak manipulation explanation, we would expect to see a trend in the direction of the hypothesized effect, or some analogy change in the high working memory performance change cases. Instead, participants’ performance on the verbal analogy task was unchanged by the training, and their performance on the visual analogy task was essentially identical to that of the control participants. The lack of any indication of change suggests that even a substantial increase in the initial working memory training effect on working memory capacity is unlikely to produce transfer effects on the analogy tasks.

Despite the correlational and experimental evidence linking working memory and analogical reasoning, it is possible that prior research may have overestimated the role of working memory capacity in analogical reasoning. Prior knowledge and strategies allow individuals to work with very complex information without overwhelming their relatively limited working memory capacity. In the case of analogical reasoning, which typically involves rich semantic knowledge and conceptual strategies for mapping deep features, learners’ prior knowledge may be more important than differences in working memory capacity. Working memory capacity might still play a role in the types of prior knowledge that learners have acquired, but in this case the connection between working memory and analogical reasoning would be an indirect one affecting long-term memory structures developed across a lifetime. This suggests that the large correlations between working memory capacity and analogical reasoning ability might be at least partially mediated by the additional general knowledge that individuals with higher working memory capacity have acquired. Future work should continue to experimentally test the mechanisms responsible for successful analogical reasoning, with particular attention to possible mediators between working memory and analogical reasoning.

## Supporting Information

Dataset S1 Dataset including participants’ pre-training and post-training scores across all reported measures.
(XLSX)
